# A joint PCR-based gene-targeting method using electroporation in the pathogenic fungus *Trichosporon asahii*

**DOI:** 10.1186/s13568-022-01431-9

**Published:** 2022-07-14

**Authors:** Yasuhiko Matsumoto, Tae Nagamachi, Asami Yoshikawa, Tsuyoshi Yamada, Takashi Sugita

**Affiliations:** 1 Department of Microbiology, Meiji Pharmaceutical University, 2-522-1, Noshio, Kiyose, Tokyo 204-8588 Japan; 2 Teikyo University Institute of Medical Mycology, 359 Otsuka, Hachioji, Tokyo 192-0395 Japan; 3 Asia International Institute of Infectious Disease Control, Teikyo University, 2-11-1, Kaga, Itabashi-ku, Tokyo, 173-8605 Japan

**Keywords:** *Trichosporon asahii*, Mutant, Electroporation, Joint PCR, Gene targeting

## Abstract

*Trichosporon asahii* is a pathogenic fungus that causes deep-seated fungal infections in immunocompromised patients. Established methods for generating gene-deficient *T. asahii* mutants exist, but the frequency of obtaining transformants by electroporation remains low. In the present study, we optimized the conditions for gene transfer by electroporation using a *ku70* gene-deficient mutant with high recombination efficiency. Introducing a DNA fragment by electroporation into *T. asahii* cells on Sabouraud dextrose agar to generate a *cnb1* gene-deficient mutant and incubating for 1 day led to the growth of approximately 100 transformants. When the incubation period was extended to 2 days or 5 days, however, only 2 or no transformants, respectively, were grown. The highest number of transformants was grown by electroporation when a square wave at 1.8 kV (9 kV/cm) was applied for 5 ms. In addition, the number of transformants increased with an increase in the length of the homologous region, and transformants did not grow when the homologous region was less than 500 base pairs. A DNA fragment was produced for deletion of the *cnb1* gene by joint PCR, and the *cnb1* gene-deficient mutant was obtained by introducing the DNA fragment by electroporation. These results indicate that DNA fragments produced by joint PCR can be used to generate gene-deficient mutants of *T. asahii* through gene transfer by electroporation.

**Graphical Abstract:**

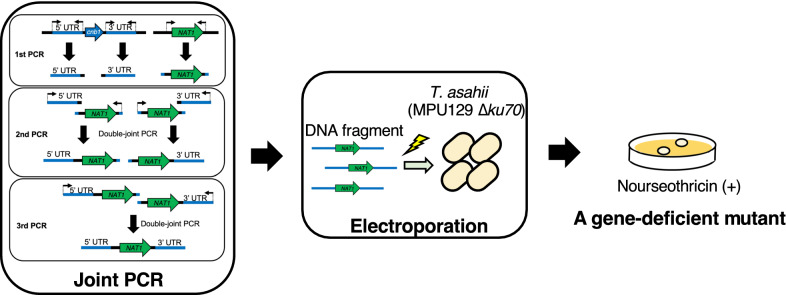

## Introduction

*Trichosporon asahii* is a basidiomycete yeast that is widely distributed in the environment and isolated from human blood, sputum, skin, feces, and urine (Sugita et al. 2001; Zhang et al. 2011; Colombo et al. 2011; Gouba et al. 2014; Cho et al. 2015). *T. asahii* is a pathogenic fungus that causes severe deep-seated fungal infections in neutropenic patients (Walsh et al. 1992, 1993; de Almeida Júnior and Hennequin 2016; Duarte-Oliveira et al. 2017). While the mortality rate of deep-seated mycosis caused by *Candida albicans* is approximately 40%, that caused by *T. asahii* is approximately 80% (Krcmery et al. 1999; Girmenia et al. 2005). *T. asahii* is resistant to echinocandin antifungals, and infectious diseases caused by *T. asahii* often occur in the patients treated with micafungin (Goodman et al. 2002; Kimura et al. 2018, 2022). Moreover, *T. asahii* strains resistant to antifungals such as amphotericin B and fluconazole have been isolated from patients (Toriumi et al. 2002; Iturrieta-González et al. 2014). Therefore, revealing the infection mechanisms and drug-resistant systems of *T. asahii* is crucial.

Phenotypic analyses using gene-deficient mutants are important for clarifying the molecular mechanisms of infection and drug resistance in *T. asahii*. We established a method for generating a gene-deficient mutant of *T. asahii* using an *Agrobacterium tumefaciens*-mediated gene transfer system (Matsumoto et al. 2021). The efficiency of genetic recombination in *T. asahii* was increased by deleting the *ku70* gene that encodes the Ku70 protein, a subunit of the non-homologous end-joining repair enzyme (Matsumoto et al. 2021). The *ku70* gene-deficient mutant exhibited the same proliferative ability in vitro and the same virulence in a silkworm infection model as the wild strain of *T. asahii* (Matsumoto et al. 2021). Therefore, the *ku70* gene-deficient mutant of *T. asahii* is useful as a parental strain for generating gene-deficient mutants by homologous recombination.

The development of simple methods for generating gene-deficient mutants requires the optimization of gene transfer methods and improved construction of gene-targeting plasmids. For deletion of a target gene by homologous recombination, a DNA fragment containing the 5'-UTR and 3'-UTR of the target gene is necessary. The DNA fragment can be produced by joint PCR without cloning using *Escherichia coli* (Yu et al. 2004). A method for producing a gene-deficient mutant of *Cryptococcus neoformans*, a closely related species of *T. asahii,* was established using a DNA fragment produced by joint PCR (Lin et al. 2015). The DNA fragment used for deletion of the target gene is amplified by PCR and introduced into *T. asahii* by electroporation. When the *ku70* gene-deficient mutant of *T. asahii* is used as the parent strain, however, the number of transformants obtained by electroporation is less than 10 (Matsumoto et al. 2021).

In the present study, we optimized the electroporation conditions for gene transfer into *T. asahii*. A gene-deficient mutant was generated using a DNA fragment produced by joint PCR under optimized conditions. Our findings suggest that a gene-deficient mutant of *T. asahii* can be efficiently generated by electroporation without gene cloning using *E. coli*.

## Materials and methods

### Reagents

Nourseothricin and G418 were purchased from Jena Bioscience (Dortmund, Germany) and Enzo Life Science, Inc. (Farmingdale, NY, USA), respectively.

### Culture of *T. asahii*

The *T. asahii* strain (MPU129 *ku70* gene-deficient mutant) used in this study was generated as previously reported (Matsumoto et al. 2021). The strain have been deposited in MPU culture collection. The *T. asahii* MPU129 *ku70* gene-deficient mutant was grown on Sabouraud dextrose agar (SDA) containing G418 (50 μg/ml) and incubated at 27 °C for 2 days.

### Preparing competent *T. asahii* cells

Competent *T. asahii* cells were prepared according to a previous report with slight modification (Matsumoto et al. 2021). The *T. asahii* MPU129 *ku70* gene-deficient mutant was spread on SDA and incubated at 27 °C for 1, 2, or 5 days. *T. asahii* cells on the agar were suspended in physiologic saline solution (2 ml), and the suspension was transferred to a 1.5 ml tube. The fungal cells were collected by centrifugation at 8000 rpm for 3 min (TOMY-MX100, TOMY Digital Biology Co. Ltd, Tokyo, Japan) and suspended by adding 1 ml of ice-cold water and centrifuged at 8000 rpm for 3 min. This washing process was repeated four times. The washed cells were suspended by adding 1 ml of 1.2 M sorbitol solution and centrifuged at 8000 rpm for 3 min. The obtained fungal cells were suspended with 0.2 ml of 1.2 M sorbitol solution as competent cells.

### Electroporation

Electroporation was performed according to a previous report with slight modification (Matsumoto et al. 2021). The PCR-amplified 5'-UTR (*cnb1*) -*NAT1*-3'-UTR (*cnb1*) fragment (180 ng/2 µl) was added to the competent *T. asahii* cells (40 µl) and placed on ice for 15 min. The suspension was added to a 0.2 cm gap cuvette (Bio-Rad Laboratories, Inc.) and electroporated (time constant protocol: 1.8 kV, 5 ms) using a Gene Pulser Xcell (Bio-Rad Laboratories, Inc.). The cells were suspended by adding 500 µl YPD containing 0.6 M sorbitol and incubated at 27 °C for 3 h. After incubation, the cells were collected by centrifugation at 10,000 rpm for 5 min, suspended in 100 µl of physiologic saline solution, and applied to SDA containing nourseothricin (300 µg/ml). The cells were incubated at 27 °C for 3 days, and the growing colonies were isolated as *cnb1* gene-deficient mutant candidates.

### Genotyping PCR

Genotyping PCR was performed according to a previous report with slight modification (Matsumoto et al. 2021). The transformants were grown on SDA containing nourseothricin (300 µg/ml). Colony PCR was performed using Primers-2 for *cnb1* genotyping (Table 1). The genome of transformants selected by the colony PCR was extracted using a Quick-DNA™ Fungal/Bacterial Miniprep Kit (Zymo Research, Irvine, CA, USA). The mutation of the genome of the transformants was confirmed by PCR using the primers shown in Table 1.

**Table 1:** Primers used in this study

Primers	Nucleic acid sequence
[Amplification of *cnb1* cassette]	
2000 bp	
F*cnb1*(2000 bp)	CCGTGATCTGCTGCACGTTCGGGTCCG
R*cnb1*(2000 bp)	CTGTTCACCTCTGGCTACGACCCCCTCCTC
1500 bp	
F*cnb1*(1500 bp)	ACGAGCCTTGCGCTGGGCCTCCT
R*cnb1*(1500 bp)	GCTCCTGCCGGTGGCTACGAGCAGCC
1000 bp	
F*cnb1*(1000 bp)	CATTGCCGCCAACCTTGAGTGCTCGGAGC
R*cnb1*(1000 bp)	GCCGCGCCCCCGCCAGCCGGGAAAC
500 bp	
F*cnb1*(500 bp)	CGCGCCGCGCTGTCCCATCGCTATTACAC
R*cnb1*(500 bp)	CATACCCGCCCCTTTCCTATGTGACTGATACC
250 bp	
F*cnb1*(250 bp)	CTGCGTTGGAGGACACGTCTACTCGCCATGA
R*cnb1*(250 bp)	ACATGTCATGATGTTCATGGAAGGGGAAGAG
[Joint PCR]	
*cnb1*(5ʹUTR)	
F*cnb1*(5ʹUTR)	ACGAGCCTTGCGCTGGGCCTCCT
R*cnb1*(5ʹUTR)(*NAT1* inf)	AGCTCACATCCTCGCAGCGCAGTGCAATCTTGGTCAGTCGGTCGTGGCC
*cnb1*(3ʹUTR)	
F*cnb1*(3ʹUTR)(*NAT1* inf)	TAGTTTCTACATCTCTTCGCGCGCACACACGGATGTGAGCGTAACGAG
R*cnb1*(3ʹUTR)	GCTCCTGCCGGTGGCTACGAGCAGCC
*NAT1*	
F*NAT1*(5ʹUTR inf)	TGACCAAGATTGCACTGCGCTGCGAGGATGTGAGCTGGAGAGC
R*NAT1*(3ʹUTR inf)	ACATCCGTGTGTGCGCGCGAAGAGATGTAGAAACTAGCTTCCTGGTTTC
*cnb1*(5ʹUTR)-*NAT1*	
F*cnb1*(5ʹUTR)	ACGAGCCTTGCGCTGGGCCTCCT
R*NAT1*(3ʹUTR inf)	ACATCCGTGTGTGCGCGCGAAGAGATGTAGAAACTAGCTTCCTGGTTTC
*NAT1*- *cnb1*(3ʹUTR)	
F*NAT1*(5ʹUTR inf)	TGACCAAGATTGCACTGCGCTGCGAGGATGTGAGCTGGAGAGC
R*cnb1*(3ʹUTR)	GCTCCTGCCGGTGGCTACGAGCAGCC
*cnb1*(5ʹUTR)-*NAT1*-*cnb1*(3ʹUTR)	
F*cnb1*(5ʹUTR)	ACGAGCCTTGCGCTGGGCCTCCT
R*cnb1*(3ʹUTR)	GCTCCTGCCGGTGGCTACGAGCAGCC
[Genotyping]	
Primers-1 for *cnb1* genotyping	
F *cnb1* gene locus	GGAGTGAAGAAGGGCAGAGAGCAACAACAGCGGT
R *cnb1* gene locus	CCGTGATCGCATGGGGCGTGCACAAAGTG
Primers-2 for *cnb1* genotyping	
F *cnb1* gene ORF	CGGCTCGGGTACGGTAGACTTCCAGGAGTTTGTCG
R *cnb1* gene ORF	AACAGGTCCTCGAGCGTCATCTGCTTGACGATGT

### Amplification of DNA fragments by joint PCR

Joint PCR was performed according to a previous report with slight modification (Lin et al. 2015). The genome of *T. asahii* MPU129 strain was used as a template to amplify the 5′-UTR (*cnb1*) and 3′-UTR (*cnb1*), and the previously generated 5'-UTR (*cnb1*)-*NAT1*-3'-UTR (*cnb1*) was used as a template to amplify the *NAT1* gene (1st PCR). Double-joint PCR was performed using the PCR products obtained by the 1st PCR to amplify 5'-UTR(*cnb1*)-*NAT1* and *NAT1*-3'-UTR(*cnb1*) (2nd PCR). The 5'-UTR (*cnb1*)-*NAT1*-3'-UTR (*cnb1*) was amplified by double-joint PCR using the PCR product obtained by 2nd PCR (3rd PCR).

## Results

### Optimization of electroporation conditions for the introduction of DNA fragments into *T. asahii*

**Fig. 1:**
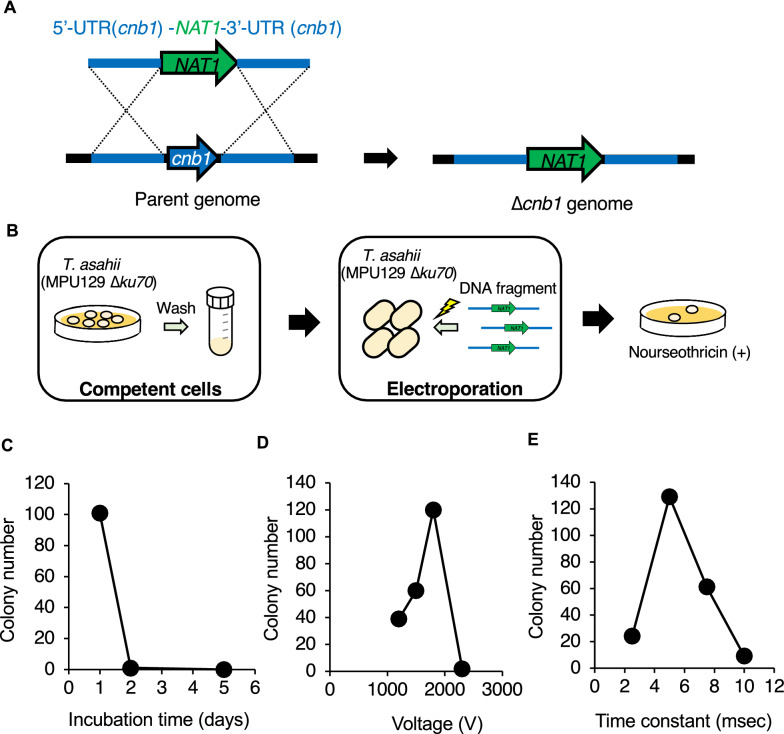
Optimization of the conditions for gene transfer by electroporation in *T. asahii*. **A** Structure of the DNA fragment for constructing the *cnb1* gene-deficient mutant and the predicted genome of the *cnb1* gene-deficient mutant. **B** Scheme for obtaining drug-resistant strains by gene transfer via electroporation. **C** Effect of the number of incubation days for preparing competent *T. asahii* cells. The *T. asahii* MPU129 *ku70* gene-deficient mutant was spread on SDA and incubated at 27 °C for 1, 2, or 5 days. The PCR-amplified 5'-UTR (*cnb1*) -*NAT1*-3'-UTR (*cnb1*) fragment (180 ng/2 µl) was added to the competent *T. asahii* cells (40 µl) and electroporated (time constant protocol: 1.8 kV, 5 ms). The number of colonies grown on SDA containing nourseothricin (300 µg/ml) was counted. **D** Effect of voltage on gene transfer by electroporation. The PCR-amplified 5'-UTR (*cnb1*) -*NAT1*-3'-UTR (*cnb1*) fragment (180 ng/2 µl) was added to competent *T. asahii* cells (40 µl) prepared by culture for 1 day and electroporated (time constant protocol: 1.2–2.1 kV, 5 ms). The number of colonies grown on SDA containing nourseothricin (300 µg/ml) was counted. **E** Effect of time constant on gene transfer by electroporation. The PCR-amplified 5'-UTR (*cnb1*) -*NAT1*-3'-UTR (*cnb1*) fragment (180 ng/2 µl) was added to competent *T. asahii* cells (40 µl) prepared by culture for 1 day and electroporated (time constant protocol: 1.8 kV, 3–10 ms). The number of colonies grown on SDA containing nourseothricin (300 µg/ml) was counted

In this study, we used a DNA fragment containing the 5'-UTR, the 3'-UTR of the *cnb1* gene, and the *NAT1* gene, a nourseothricin resistance gene (Fig. 1A). Increasing the gene transfer efficiency is necessary to optimize the conditions for the preparation of competent cells and electroporation (Fig. 1B). Proliferative phase cells often have higher competency than stationary phase cells and are used as competent cells for gene transfer in yeast (McGlincy et al. 2021). *T. asahii* cells were spread on SDA and incubated for 1, 2, or 5 days, respectively, and collected to prepare competent cells. When a DNA fragment was introduced by electroporation into a competent cell that had been cultured for 1 day to generate a *cnb1* gene-deficient mutant, approximately 100 colonies grew on SDA containing nourseothricin (Fig. 1C). On the other hand, only 2 colonies grew when the incubation period was 2 days, and no colonies were observed when the incubation period was 5 days (Fig. 1C). This finding suggests that the competency of *T. asahii* is decreased by longer incubation.

Next, we optimized the electroporation conditions. Approximately 120 colonies were grown on SDA containing nourseothricin at 1.8 kV (Fig. 1D). In the other conditions, the number of colonies was lower than when the voltage was 1.8 kV (Fig. 1D). The highest number of colonies grew on SDA containing nourseothricin when the time constant was 5 ms (Fig. 1E). These results suggest that 1.8 kV (9 kV/cm) for 5 ms is the optimal condition for electroporation to introduce DNA fragments into the *T. asahii* MPU129 *ku70* gene-deficient mutant.

**Fig. 2:**
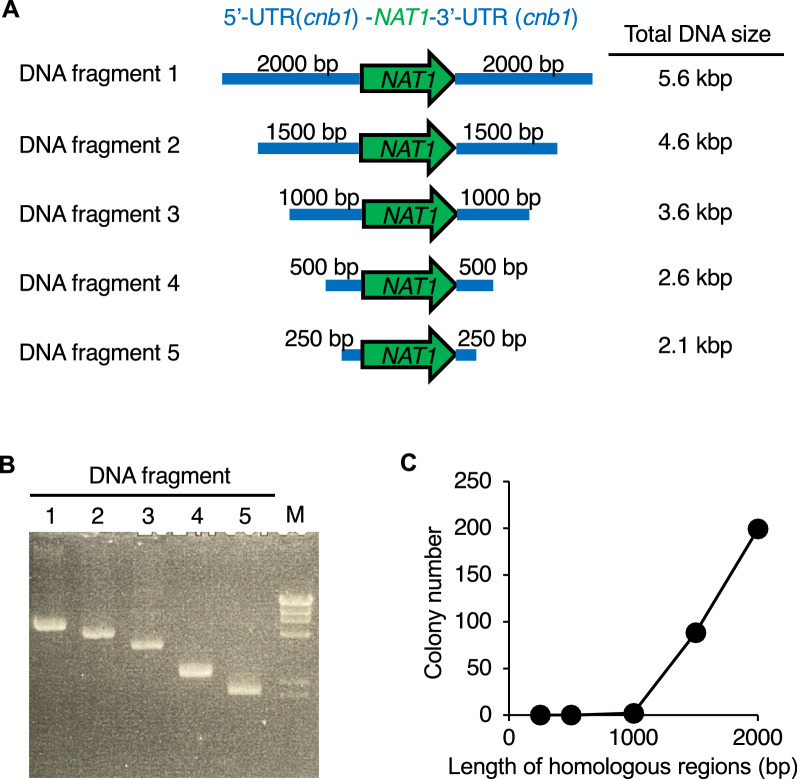
Effect of the length of homologous regions for gene transfer by electroporation in *T. asahii*. **A** Illustration of DNA fragments used in this study. **B** Electrophoresis of DNA fragments amplified by PCR. **C** The PCR-amplified 5ʹ-UTR (*cnb1*) -*NAT1*-3ʹ-UTR (*cnb1*) fragments were added to the competent *T. asahii* cells prepared by culture for 1 day and electroporated (time constant protocol: 1.8 kV, 5 ms). The number of colonies grown on SDA containing nourseothricin (300 µg/ml) was counted

More than 500 base pairs of homologous regions are required for gene deletion by double crossover in the filamentous fungi (Yu et al. 2004). On the other hand, DNA fragments, including longer homologous regions, are difficult to produce by joint PCR. We then examined the effect of the length of the homologous region on the efficiency of genetic recombination in *T. asahii*. DNA fragments with different lengths of homologous regions were amplified by PCR (Fig. 2A, B). The number of colonies grown on SDA containing nourseothricin increased as the length of the homologous region increased (Fig. 2C). Colonies did not grow on SDA containing nourseothricin when the homologous region was less than 500 bp (Fig. 2C). The results suggest that a homologous region longer than 1500 bp enhanced the efficiency of gene transfer into the *T. asahii* cells.

**Fig. 3:**
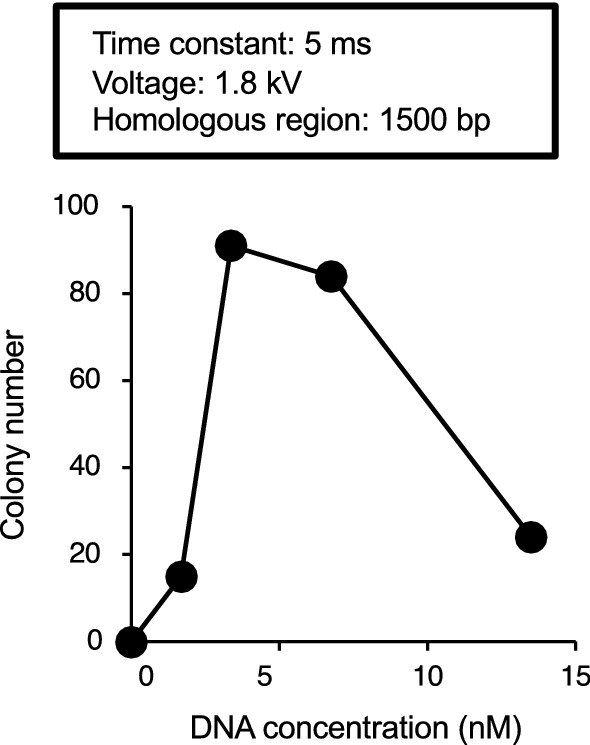
Effect of the DNA concentration for gene transfer by electroporation in *T. asahii*. The DNA fragment 2 (0–13.5 nM) was added to competent *T. asahii* cells prepared by culture for 1 day and electroporated (time constant protocol: 1.8 kV, 5 ms). The number of colonies grown on SDA containing nourseothricin (300 µg/ml) was counted

Next, we examined the optimal concentration of DNA fragments for electroporation. When competent cells were electroporated with DNA fragment 2 having a homologous region of 1500 bp, more than 80 colonies grew at 3.4 and 6.8 nM (Fig. 3). In other conditions, the number of colonies was less than 30 (Fig. 3). These results suggest that a DNA fragment concentration of approximately 3.4–6.8 nM is optimal for gene transfer by electroporation.

### Generation of a gene-deficient mutant using DNA fragments produced by joint PCR

**Fig. 4:**
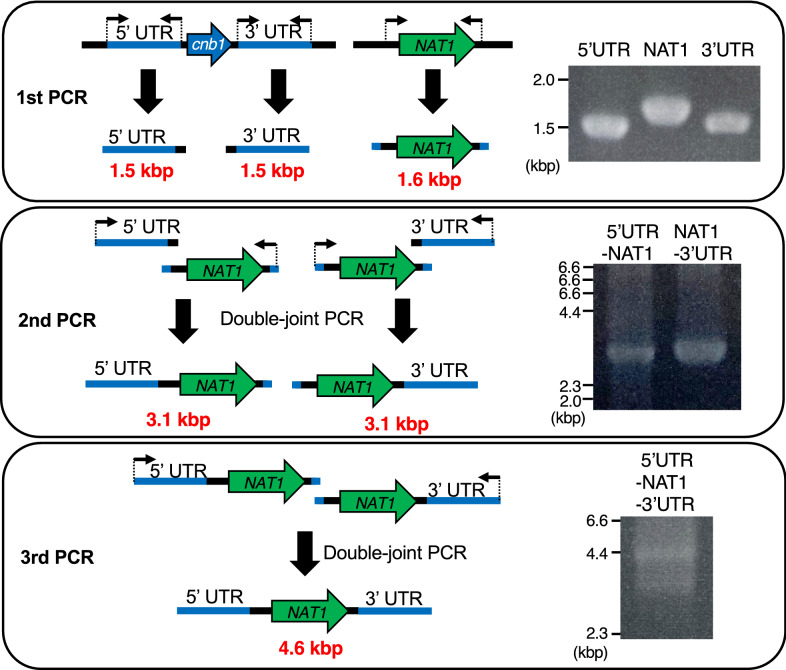
Construction of DNA fragment for gene targeting by joint PCR. Scheme of constructing DNA fragments for gene targeting by joint PCR to generate *cnb1* gene-deficient mutants and electrophoresis of DNA fragments amplified by PCR or joint PCR

**Fig. 5:**
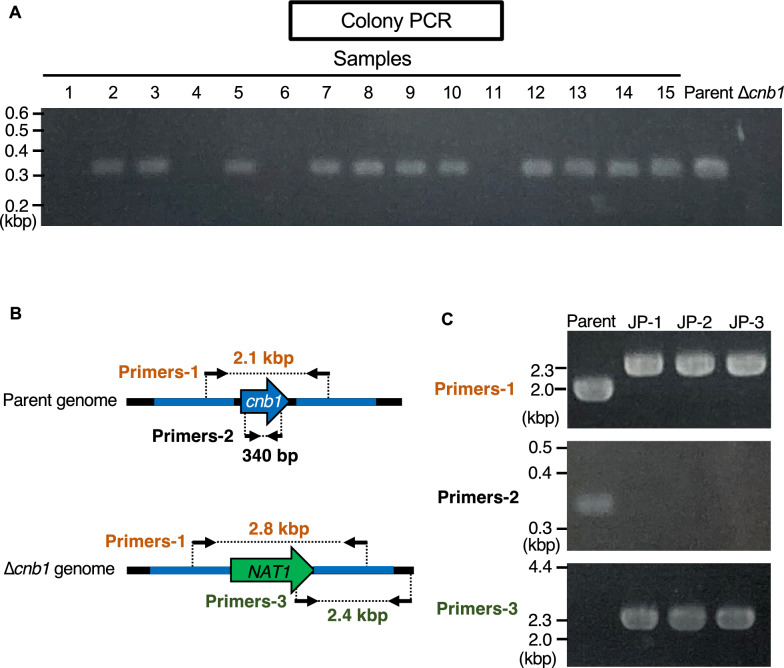
Generation of *cnb1* gene-deficient mutant using DNA fragment produced by joint PCR. **A** DNA fragment 2 produced by joint PCR was added to competent *T. asahii* cells prepared by culture for 1 day and electroporated (time constant protocol: 1.8 kV, 5 ms). Colony PCR was performed on colonies (samples 1–15) grown on SDA containing nourseothricin (300 µg/ml). **B** Location of the primers for confirming the genome structure of the *cnb1* gene-deficient candidate by PCR. **C** Confirmation of the *cnb1* gene-deficiency of the *cnb1* gene-deficient candidate by PCR using extracted genome DNA

Joint PCR is useful for obtaining DNA fragments without cloning in *E. coli* (Yu et al. 2004). We examined whether a *cnb1* gene-deficient mutant could be obtained using DNA fragments produced by joint PCR. Each joint PCR step amplified DNA fragments with the predicted size, and a DNA fragment for generating the *cnb1* gene-deficient mutant was obtained (Fig. 4). Nourseothricin-resistant strains were obtained by introducing the DNA fragment into *T. asahii* by electroporation, which was optimized in this study. Among the 45 nourseothricin-resistant strains, 8 strains were deficient for the *cnb1* gene (Fig. 5, Table 2). These results suggest that the *cnb1* gene-deficient mutants of *T. asahii* were generated using DNA fragments amplified by joint PCR.

**Table 2:** Efficiency of homologous replacement in *cnb1* gene region

DNA fragment	Length of homologous regions (bp)	Total transformants	Homologous replacement (∆*cnb1*)	Efficiency (%) (∆*cnb1*/total transformants)
Joint PCR product	1500	45	8	18%
DNA fragment 1^a^	2000	21	4	19%

^a^Data are cited from Matsumoto et al.(Matsumoto et al. 2021)

## Discussion

Here, we developed a method to establish a *cnb1* gene-deficient mutant of *T. asahii* using PCR-amplified DNA fragments without gene cloning in *E. coli*. Using this method, it is theoretically possible to obtain a gene-deficient mutant of *T. asahii* within 1 week. Further experiments are needed to determine whether strains deficient in other genes can be obtained under these experimental conditions.

Optimizing the preparation of competent cells and electroporation conditions is very important for improving gene transfer (Edman and Kwon-Chung 1990; Wang 2018). The transformants in this study were obtained by introducing a drug-resistant gene via homologous recombination and non-homologous end-joining repair. In a previous study, we used competent cells cultured for 2–3 days, and the number of transformants obtained was less than 10 (Matsumoto et al. 2021). Competent cells incubated for 1 day had higher competency than those incubated for 2 days.

For generating gene-deficient mutants by electroporation, longer homologous regions should be used to increase the recombination efficiency (Ueno et al. 2007). On the other hand, the use of long DNA fragments decreased the gene transfer efficiency (Lamichhane et al. 2015). Moreover, long DNA fragments are difficult to produce by joint PCR (Bryksin and Matsumura 2010). Therefore, optimizing the length of the homologous regions is important toward increasing the efficiency of obtaining gene-deficient mutants. In *T. asahii*, a 1500-bp homologous region is needed to obtain gene-deficient mutant candidates by electroporation at a ratio of approximately 20%. The efficiency of homologous replacement in the *cnb1* gene region by joint PCR products in this study is similar to that in a previous study in which PCR products were amplified using a purified targeting plasmid (Matsumoto et al. 2021). Therefore, joint PCR products are useful for generating gene-deficient mutants in *T. asahii* by gene transfer via electroporation. A DNA fragment amplified by joint PCR for deletion of a target gene can be used to generate *cnb1* gene-deficient mutants in *T. asahii*. This method enabled us to produce DNA fragments for the deletion of target genes more rapidly and conveniently than the conventional method of producing DNA fragments using *E. coli*. In a future study, we will generate gene-deficient mutants of *T. asahii* established in this study and analyze their pathogenicity functions.

In conclusion, we established a simple method for generating gene-deficient mutants in *T. asahii* via electroporation. The method might contribute to uncovering the molecular mechanisms of infection and drug resistance in *T. asahii*.

## Data Availability

All data are presented in figures and tables within this article. Any material used in this study will be available for research purposes upon request.
